# Accuracy of digital tooth preparations recorded using the plaster model scanning technique and two silicone impression scanning techniques

**DOI:** 10.1016/j.heliyon.2024.e40477

**Published:** 2024-11-17

**Authors:** Yuyin Shen, Zhicheng Gong, Jue Wang, Shuobo Fang

**Affiliations:** a Department of Dental Technology, Shanghai Stomatological Hospital & School of Stomatology, Fudan University, Shanghai, China; b Department of Prosthodontics, Shanghai Stomatological Hospital & School of Stomatology, Shanghai Key Laboratory of Craniomaxillofacial Development and Diseases, Fudan University, Shanghai, China

**Keywords:** Digital impression technique, Fixed restorations, Tooth preparation

## Abstract

**Objective:**

To compare the accuracy of digital tooth preparations recorded using the plaster model scanning technique and two silicone impression scanning techniques.

**Materials and methods:**

A maxillary resin model with incisor and first molar abutment preparations was used as the experimental model and scanned to serve as the gold standard. Three groups of digital models were generated, with 10 models in each group. Group 1 comprised 10 silicone impressions of the experimental model and were scanned using the 3Shape Trios3 intraoral scanner. Group 2 comprised 10 impressions covered with a scanning spray and scanned using the 3Shape D2000 extraoral scanner. Group 3 comprised plaster models made from the 10 impressions and scanned using the 3Shape D2000 extraoral scanner. Root mean square values were obtained by comparing the scanning data of each group with the gold standard using the Geomagic software. The one-way analysis of variance and paired student's *t*-tests were used to analyse the root mean squares; the significance level was set at 0.05.

**Results:**

No statistically significant difference was observed in the root mean squares of the non-prepared teeth among the three groups (P = 0.12). The mean root mean squares of Groups 1, 2, and 3 were 0.11 ± 0.03 mm, 0.07 ± 0.03 mm, and 0.10 ± 0.05 mm, respectively. A statistical difference was observed in the incisor area among the three groups (P < 0.05), wherein Group 2 showed the lowest root mean square value (0.06 ± 0.02 mm). No significant difference was observed in the first molar area among the three groups (P = 0.142).

**Conclusions:**

Digital tooth preparations models obtained from silicone impressions by extraoral scanning showed similar accuracy to intraoral scanning and plaster model scanning. This indicates that the clinical workflow of taking silicone impressions and scanning with an extraoral scanner is an acceptable and convenient choice.

## Highlights

• This study was designed to explore whether it is possible to create a digital model by scanning silicone impressions instead of making plaster models in fixed restorations. The results showed that digital models obtained from silicone impressions by extraoral scanning showed similar accuracy to intraoral scanning and plaster model scanning. This indicates that the clinical workflow proposedis an acceptable choice.

## 1 Introduction

Nowadays, computer aided design/computer aided manufacturing (CAD/CAM) technology is widely used to manufacture fixed restorations. Fixed restorations are designed using a CAD system and milled or printed using a CAM machine [1].

To create a digital model for the CAD step, two scanning strategies can be employed, namely the direct and indirect scanning methods [1]. The direct scanning method uses an intraoral scanner to obtain a digital impression. The indirect scanning method involves making a plaster model from the impression and scanning it using an extraoral scanner. Although intraoral scanning is comfortable for patients [2] and has a highly reliable accuracy [3,4], it has certain limitations. First, intraoral scanners have difficulties in scanning narrow and deep subgingival margins [5]. Hence, gingival retraction is needed to expose the subgingival preparation for accurate scanning. Moreover, its accuracy is affected by the physical properties of the object's surface because different materials detect and reflect light differently [6,7]. An increase in the scanning range can reduce the scanning accuracy [8]. Finally, the operator's skills and decisions are important factors affecting scanning accuracy. Thus, operators must have a thorough understanding of the scanning equipment and procedures to perform the scanning correctly [9].

In the traditional manufacturing process, the accuracy of the silicone impression is directly related to the success of a fixed restoration [10]. Many studies have shown that the contact angle of silicone impression materials is > 90°, indicating that they are hydrophobic impression materials with poor surface wettability [[11], [12], [13]]. After obtaining the silicone impression, a plaster model is created. Related studies have suggested that poor surface wettability may lead to insufficient flow of the liquid plaster into the impression surface, resulting in voids in the plaster models, especially at the edges of the prepared teeth, pinholes, undercut grooves, and other areas, affecting the accuracy and precision of the restoration [14]. Furthermore, the plaster expands upon setting [15], and the volume of the model increases if the water/powder ratio is less or the mixing time is longer than recommended. Additionally, various errors are superimposed because of the unavoidable influence of human factors during the model perfusion process, thus affecting the fit of the restorations.

The evolution of fixed restoration manufacturing has made it possible for dental technicians to design fixed restorations by solely relying on digital models; hence, a plaster model seems no longer necessary in the manufacturing process. Using an extraoral scanner to scan impressions and generate digital models eliminates the need to create plaster models and simplifies the manufacturing process. Although a scanning spray is required during impression scanning [16] to ensure uniform light reflection [17], studies have shown that the coating thicknesses of scanning sprays are within an acceptable clinical range [18].

According to Persson et al. [19], impressions and stone replicas can be digitised repeatedly with high reliability. Kim et al. [20] reported that digital models obtained through silicone impression scanning exhibited higher accuracy than those obtained from alginate impressions. However, the literature on the feasibility of using intraoral scanners for impression scanning is limited. Moreover, it is unclear whether digital models obtained by impression scanning can replace plaster models.

Therefore, this study aimed to compare the accuracy of tooth preparations recorded using plaster model scanning and two digital silicon impressions and explore whether it is possible to create a digital model by scanning silicone impressions instead of making a plaster model during the manufacturing process of fixed restorations and to optimize the manufacturing process. The null hypothesis is that there is no significant difference in the mean values of the outcome variables among the three techniques.

## 2 Materials and methods

A maxillary full-mouth dental model composed of synthetic resins was selected, wherein the maxillary left incisor and right first molar were prepared as the abutment teeth. The abutment teeth showed the following parameters: occlusal reduction, 1.5 mm; chamfer margin, 1.0 mm; and axial wall angle, 6°. The resin model was scanned using an extraoral scanner (D2000, 3Shape) as the gold standard. The scanner was calibrated according to the manufacturer's instructions, and the scanner settings were adjusted for high-resolution digitisation.

The sample size for each group was calculated using G∗Power version 3.1.9.7 (Heinrich-Heine-Universität, Düsseldorf). One-way analysis of variance (ANOVA) was used to analyse differences among the groups. The effect size was 0.6, the significance level was 0.05, and the power level was 0.8. Finally, a sample size of 10 was set for each group.

**Fig. 1:**
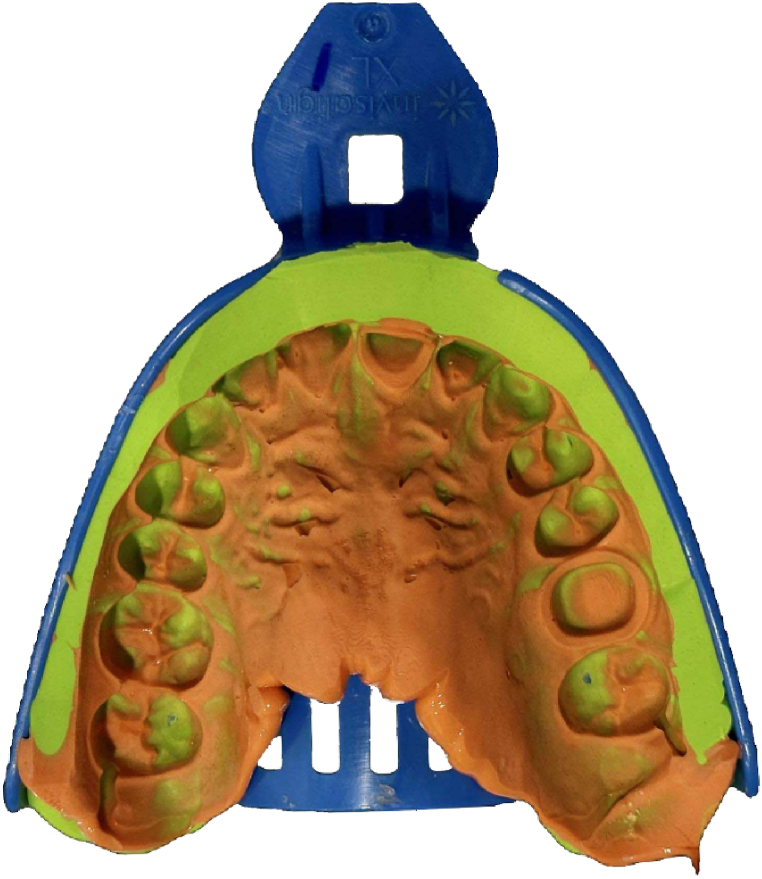
Silicone impression.

Ten plastic custom trays with small holes (plastic custom trays XL, Invisalign) and a corresponding quantity of silicone impression material (VariotimeLight Flow and Monophase, Heraeus Kulzer GmbH) were prepared to take impressions. The silicone impression material was syringed onto the surfaces of the two abutment preparations. A plastic custom tray was filled with a low-flow impression material and placed on the resin model. The impression tray was separated from the resin model according to the setting time of the impression material specified by the manufacturer. Excess material was trimmed away using a scalpel to expose the margins of the preparations. In total, 10 silicone impressions were obtained for each group (Fig. 1).

**Fig. 2:**
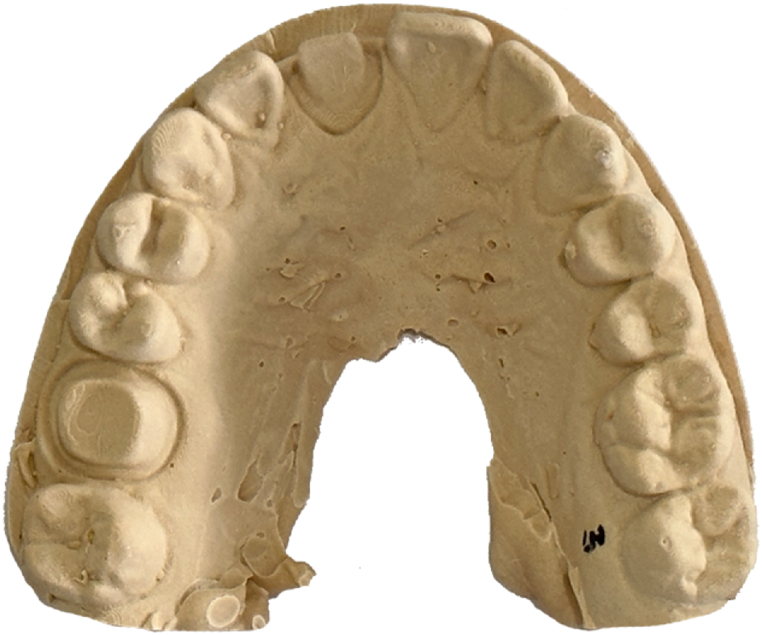
Plaster-model made by the silicone impression.

**Fig. 3:**
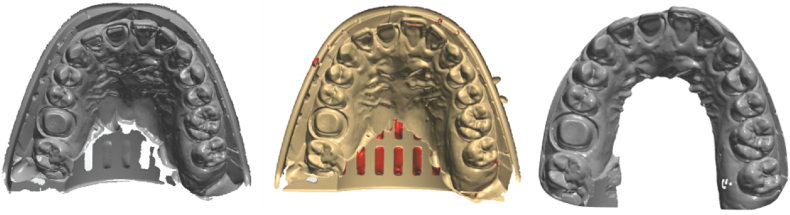
(a) Silicone impression scanned by intraoral scanner; (b) silicone impression scanned by extraoral scanner; (c) plaster-model scanned by extraoral scanner.

In Group 1, the 10 silicone impressions were scanned using an intraoral scanner (Trios3, 3Shape). In Group 2, the silicone impressions were covered with a scanning spray (500600, Dentaco) and scanned using an extraoral scanner (D2000, 3Shape). To obtain digital silicone impressions, the impression scan mode was selected in the software. In Group 3, the silicone impressions were covered with the same spray as in Group 2 and then placed under running water to rinse the scanning spray. Plaster (Silky-Rock, Whip Mix) was mixed according to the manufacturer's instructions and poured onto the silicone impressions, and 10 plaster models were obtained (Fig. 2). The extraoral scanner (D2000, 3Shape) was used to scan the plaster models to obtain the digital models. All procedures were executed 1 day after the impressions were taken as per the recommended times specified in the product documentation. All digital models obtained using the three methods were exported as stereolithography (STL) files (Fig. 3(a)–(c)).

**Fig. 4:**
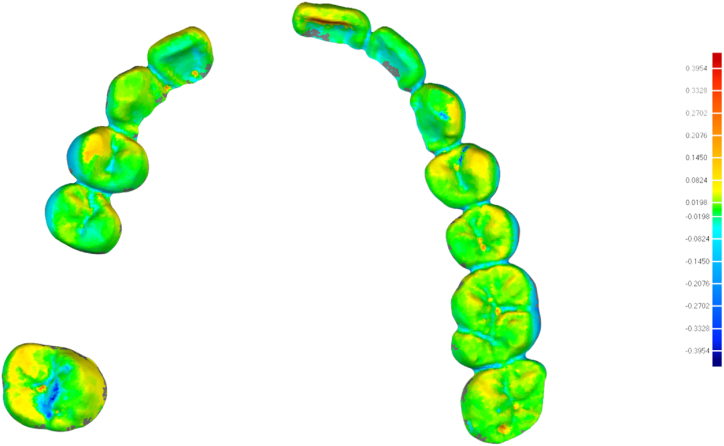
3D comparison between a digital model and the golden standard model.

**Fig. 5:**
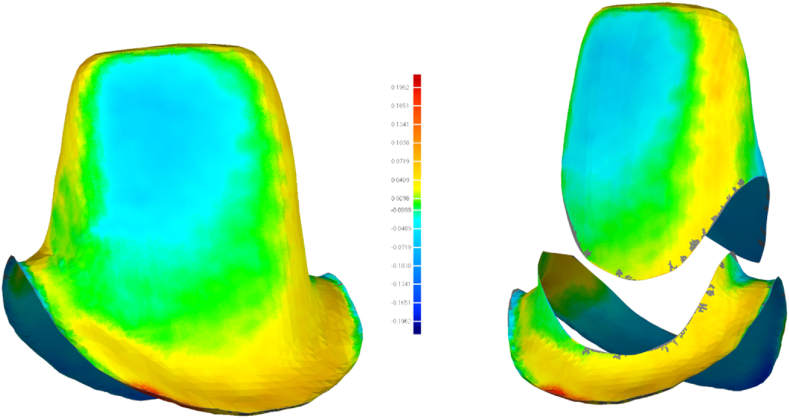
3D comparison between the incisal area in a digital model and the golden standard model.

**Fig. 6:**
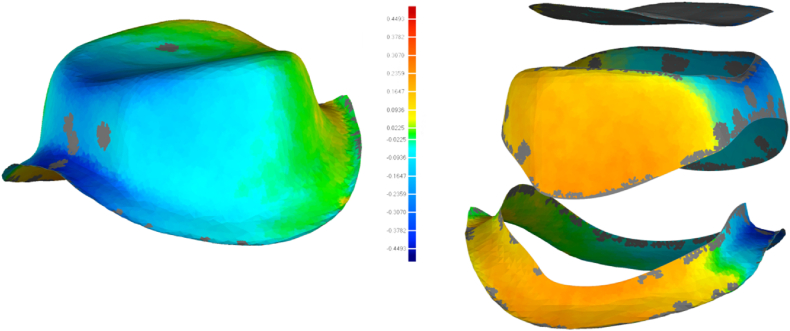
3D comparison between the molar area in a digital model and the golden standard model.

The STL files were imported into the matching software (Geomagic Control X 64, Oqton) to evaluate the accuracy of the digital models. Residual teeth were chosen as the common area, and the “best-fit alignment” option was used to superimpose the STL file of each group on the gold standard STL file. The “3D compare” feature of the software was used to evaluate the overall accuracy of the measured data (Fig. 4). The digital models were trimmed, leaving only the two prepared teeth. Furthermore, “3D compare” was used to evaluate the accuracy of recording the tooth preparations. For further analysis, the incisor preparation was segmented into the cervical (2 mm above the margin) and incisal parts, while the molar preparation was segmented into the cervical (2 mm above the margin), middle, and occlusal parts. The “3D compare” feature was used to evaluate the accuracy of each part. These steps were repeated for all digital models (Fig. 5, Fig. 6).

The root mean square (RMS) represents the accuracy of the digital models. Accuracy was also represented in the form of a colour scale. The red area indicated that the experimental group image surface was higher than the gold standard image surface, and the RMS was positive. The blue area indicated that the experimental group image surface was lower than the gold standard image surface, and the RMS was negative. The smaller the absolute value of the RMS, the closer it was to the middle chromatographic segment (green area), representing the best match between the experimental group and the gold standard.

All data were collected, and statistical analyses were performed using IBM SPSS Statistics version 27. The ANOVA, paired student's *t*-test, and Bonferroni post-hoc test were used to analyse the RMS values. The significance level was set at 0.05.

## 3 Results

No statistical difference was observed in the RMS values of the non-prepared dentition among the three groups (P > 0.05). The mean RMS values of Groups 1, 2, and 3 were 0.11 ± 0.03 mm, 0.07 ± 0.03 mm, and 0.10 ± 0.05 mm, respectively (Table 1).

**Table 1:** Comparison of the average RMS among the Groups.

Group	Number	Mean (mm)	SD (mm)	P-value
1	10	0.11	0.03	0.12
2	10	0.07	0.03	
3	10	0.10	0.05	

RMS: Root mean square; SD: Standard deviation; Group 1: silicon impression + intraoral scanner; Group 2: silicon impression + extraoral scanner; Group 3: plaster model + extraoral scanner.

A statistically significant differences was observed in the incisor area among the three groups (P < 0.05), with Group 2 showing the lowest RMS value at 0.06 ± 0.02 mm. No statistically significant difference was observed in the first molar area among the three groups (P > 0.05) (Table 2).

**Table 2:** Comparison of the RMS of the incisor and first molar preparations among the Groups.

Tooth location	Group	Mean (mm)	SD (mm)	P-value	Bonferroni post-hoc test
Incisor	1	0.08	0.02	0.017	Group 1 > Group 2∗ Group 2 < Group 3∗
	2	0.06	0.02		
	3	0.09	0.03		
First Molar	1	0.14	0.08	0.516	–
	2	0.11	0.07		
	3	0.14	0.08		

RMS: Root mean square; SD: Standard deviation; Group 1: silicon impression + intraoral scanner; Group 2: silicon impression + extraoral scanner; Group 3: plaster model + extraoral scanner.

Significance level: ∗P < 0.05, ∗∗P < 0.01.

Evaluation of the differences between the different parts of the prepared incisors and first molars showed no statistically significant difference (P > 0.05). The RMS values of the cervical and incisal parts of the incisor were 0.07 ± 0.02 mm and 0.07 ± 0.03 mm, respectively. The RMS values of the cervical, middle, and occlusal parts of the first molar were 0.13 ± 0.05 mm, 0.11 ± 0.04 mm, and 0.16 ± 0.14 mm, respectively (Table 3).

**Table 3:** Comparison of the RMS among different parts of the prepared incisors and first molars.

Tooth Location	Part	Mean (mm)	SD (mm)	P-value
Incisor	Cervical	0.07	0.02	0.225
	Incisal	0.07	0.03	
First Molar	Cervical	0.13	0.05	0.142
	Middle	0.11	0.04	
	Occlusal	0.16	0.14	

RMS: Root mean square; SD: Standard deviation.

## 4 Discussion

The current findings show significant differences in the RMS values among the three scanning techniques for digital tooth preparations in the incisor area. The deviation in the incisor preparation in silicone impressions scanned using the extraoral scanner was the lowest; however, no significant differences were observed in the first molar area. Hence, the null hypothesis was rejected for the incisor area and accepted for the molar area. The results also indicated that digital tooth preparations from digital silicone impressions scanned by an extraoral scanner revealed superior accuracy in the incisor area, suggesting that it is the recommended way to scan silicone impressions for the incisor area.

This study found no statistical difference in the RMS values of the different parts of the incisors and molars among the three groups, suggesting that the accuracy of the cervical part in the digital impressions is acceptable for clinical application. The accuracy of the cervical part in a dental model is one of the factors affecting the marginal fit on which clinicians focus [21]. The mean RMS value of the cervical part of the incisors and first molars was 0.07 ± 0.02 mm and 0.13 ± 0.05 mm, respectively. These findings are consistent with those observed in previous studies [22,23]. However, a previous study reported inconsistent results, in which the difference was attributed to the fact that it was difficult for the light to reach the narrow and deep part of the impression, and these parts were not scanned completely [19]. The use of different brands of scanners could have also led to inconsistencies in the results [24]. The accuracy of the other parts of the teeth, except for the cervical part of tooth preparation, determines the gap for the luting agent. Using CAD software, dental technicians can set different gap values for different parts of the prepared tooth according to the digital model type to compensate for errors in the digital model.

No statistically significant differences were observed in the RMS values of the non-prepared dentition among the three groups. This suggests that it is feasible to scan silicone impressions directly during the manufacturing process using CAD/CAM technology without making plaster models. Scanning silicone impressions directly has some unique advantages. It reduces costs, promotes process efficiency, and avoids the risk of damage to the plaster model during transportation. Dentists or nurses can scan the silicone impression using an intraoral or extraoral scanner after the impression is taken. Following this, they can send the digital model information to the dental laboratory, without needing to make plaster model and transporting it. One main difference between CAD/CAM technology and the traditional technology used for fixed restorations is data collection and storage. Dental technicians can design fixed restorations based on the information obtained from digital models. However, in a study comparing the margin fit of crowns made by scanning silicone impressions and by scanning stone replicas, the marginal discrepancy in the latter was smaller than that in the former. This difference could be attributed to the type of scanner, the scanning powder, and the model used [23].

After the maxillary and mandibular impressions are obtained, an occlusal relationship should be added to the digital model to establish the virtual dentition. Two methods have been established to determine the virtual occlusal relationships. Occlusal registration scanning is one such method. First, occlusal records are acquired using occlusal registration materials intraorally. Both sides of the occlusal record are then scanned to obtain the occlusal surface morphology of the upper and lower dental arches. Finally, the occlusal surface morphology is used to register the upper and lower dental arches or the occlusal record is placed on a single-jaw dental cast for scanning, followed by registration with the opposing dental arch [25,26]. The other method is the buccal surface registration method, which is widely used in intraoral scanning. The buccal side scanning data of the dental arch are set as reference images, and the separately scanned upper and lower dental arches data are registered with it to reconstruct the static relationship between the upper and lower dental arches [25,27,28].

A limitation of this study is that scannable impressions were not used. García-Martínez et al. found that scannable impressions could be digitalised with high precision and trueness [29]. Kalantari et al. reported that restorations fabricated from scannable impression material and conventional silicone material showed a similar marginal fit [22]. Besides, the best-fit algorithm used in this study may have underestimated the deviation because it induces uniform distribution of the deviation across the arch [30,31]. It would also be interesting to explore whether silicone impression scanning could be applied to different restoration types. Therefore, further clinical studies should be designed and conducted to explore this.

## 5 Conclusions

Scanning of silicone impressions using the extraoral scanner showed similar accuracy to intraoral scanning and plaster model scanning. Thus, the clinical workflow of taking silicon impressions and scanning them using an extraoral scanner is an acceptable and convenient choice.

## CRediT authorship contribution statement

**Yuyin Shen:** Writing – original draft, Software, Formal analysis, Data curation. **Zhicheng Gong:** Supervision, Project administration, Funding acquisition, Conceptualization. **Jue Wang:** Validation, Supervision, Resources, Project administration, Funding acquisition. **Shuobo Fang:** Writing – review & editing, Software, Formal analysis, Data curation.

## Data availability

Data will be made available on request.

## Funding

This work was supported by the Science and technology talent project of Shanghai Stomatological Hospital & School of Stomatology (SHH-2022-YJ-B02), Science and technology fund project of Shanghai Stomatological Hospital & School of Stomatology (SSH-2023-08).

## Declaration of competing interest

The authors declare that they have no known competing financial interests or personal relationships that could have appeared to influence the work reported in this paper.
